# Effect of BALF-based mNGS on clinical outcomes of immunocompromised subjects with opportunistic pulmonary infections: a multicenter propensity score-matched study

**DOI:** 10.3389/fcimb.2025.1724935

**Published:** 2026-01-07

**Authors:** Shiying Guo, Linlin Wang, Xiaoyan Sai, Suping Tang, Jiajia Wang, Anqi Wang, Dong Qiu, Shuguang Han, Yan Wu, Cheng Chen

**Affiliations:** 1 Department of Respiratory and Critical Medicine, The First Affiliated Hospital of Soochow University, Suzhou, China; 2 Department of Respiratory and Critical Medicine, The First People’s Hospital of Taicang, Suzhou, China; 3 Department of Respiratory and Critical Medicine, The Affiliated Wuxi People’s Hospital of Nanjing Medical University, Wuxi, China; 4 Department of Respiratory and Critical Medicine, Central Hospital of Jiangnan University, Wuxi, China

**Keywords:** BALF, clinical outcomes, immunocompromised, mNGS, propensity score matching

## Abstract

**Background:**

Metagenomic next-generation sequencing (mNGS) is a promising tool for pathogen detection. However, its clinical utility in detecting opportunistic pulmonary infections of immunocompromised patients remains controversial.

**Methods:**

This multicenter retrospective study involving 162 immunocompromised patients with opportunistic pulmonary infections was conducted across four respiratory centers. The enrolled patients were divided into the conventional microbiological tests (CMT) group and the mNGS group based on whether mNGS of BALF was performed after admission. Propensity score-matching (PSM) was adopted to minimize selection bias, and sensitivity analysis confirmed the robustness. The primary outcomes were >30% improvement in oxygenation index (OI) at 7 days post-admission and clinical improvement by day 14 as assessed with the WHO 7-category ordinal scale. Secondary outcomes included 21-day mortality, incidence of septic shock during hospitalization, and pathogen detection rate.

**Results:**

Among the 110 patients who underwent mNGS, the results prompted modifications to the antibiotic therapy in 89 patients (80.9%), encompassing both escalation and de-escalation of therapy. The remaining 52 patients received only CMT. After the PSM, 41 matched pairs were further analyzed. Compared to the CMT group, OI improvement >30% on day 7 was more frequent in the mNGS group (41.5% vs. 9.8%, P = 0.001). Clinical improvement on day 14 in the mNGS group was higher than in the CMT group (36.6% vs. 9.8%, P = 0.004). Additionally, BALF mNGS was associated with decreased 21-day mortality (7.3% vs. 34.1%; P = 0.003) in patients with opportunistic pulmonary infections, while showing no significant association with reduced incidence of septic shock during hospitalization. Moreover, the causative pathogen detection rate was significantly higher in the mNGS group compared to the CMT group (97.6% vs. 22.0%, P<0.001), demonstrating the superior diagnostic yield of mNGS.

**Conclusion:**

Our study indicated that early BALF mNGS testing upon admission was associated with improved OI up to day 7, clinical improvement on day 14, and decreased 21-day mortality. These benefits are likely facilitated by the higher diagnostic yield of mNGS and its direct impact on guiding targeted antibiotic therapy.

## 1 Introduction

The number of non-AIDS and non-solid tumor immunocompromised patients has significantly increased, mainly attributed to the progression in immunosuppressive therapy for autoimmune diseases, as well as an increase in solid organ transplantation and hematologic malignancies (Kunihiro et al., 2019). Compared with immunocompetent individuals, immunocompromised patients are more likely to develop severe pulmonary infections, which have a high risk of acute respiratory failure and lead to higher mortality rates (Azoulay et al., 2019; Shahani et al., 2017). Accurate pathogen identification is essential for effective antimicrobial stewardship in this vulnerable population (Azoulay et al., 2020). Conventional microbiological tests (CMT) include microbial culture, molecular testing, and antigen-antibody testing. The limited sensitivity of conventional culture methods, particularly for fastidious pathogens, often hinders accurate microbiological diagnosis in clinical practice (Miao et al., 2018). Despite the high sensitivity and specificity of polymerase chain reaction (PCR), primers need to be designed according to the predicted pathogen (Liu et al., 2024). A kind of pathogen detection method that has the advantages of being faster and more sensitive than CMT in these patients is needed.

Metagenomic next-generation sequencing (mNGS) is an advanced microbial detection technology that uses NGS methods to detect nucleic acids in samples, enabling the comprehensive identification of bacteria, viruses, fungi, and parasites (Liu and Ma, 2024). Due to its higher sensitivity in pathogen detection compared to CMT and reduced interference from prior antibiotic exposure, mNGS has emerged as a valuable diagnostic tool for lower respiratory tract infections (LRTIs) (Diao et al., 2022; Miao et al., 2018). Although previous studies have demonstrated that mNGS has superior diagnostic performance for pathogens in immunocompromised patients with pulmonary infections (Chen et al., 2022; Qian et al., 2020), reports about the association of mNGS and prognosis in this vulnerable population remain limited. Furthermore, since mNGS indiscriminately detects all microbial nucleic acids, it may also identify respiratory colonizers or environmental contaminants, potentially leading to false-positive results, which makes the clinical application of BALF mNGS disputable.

This multicenter, propensity score-matched study was conducted at four respiratory centers between January 2022 and December 2024. It analyzed the diagnostic performance of BALF mNGS and its impact on clinical outcomes of immunocompromised patients with opportunistic pulmonary infections, aiming to provide more accurate guidance for the diagnosis and prognosis for this vulnerable population.

## 2 Methods

### 2.1 Study design and population

Between January 2022 and December 2024, 162 immunocompromised patients with opportunistic pulmonary infections were retrospectively recruited from four respiratory centers across China. Patients included were divided into the CMT group and the mNGS group according to whether mNGS of bronchoalveolar lavage fluid (BALF) was performed following hospital admission. Patients in the CMT group underwent CMT exclusively, while those in the mNGS group received both CMT and mNGS testing. This study was approved by the Institutional Review Boards of the First Affiliated Hospital of Soochow University (2023-074).

For inclusion in this study, patients were required to meet all of the following criteria: (1) patients with a diagnosis of autoimmune disease or solid organ transplantation or hematologic malignancies; (2) immunocompromised host with any of the following present: a. receiving corticosteroid therapy with a dose ≥20 mg prednisone or equivalent daily for ≥14 d or a cumulative dose >600 mg of prednisone (Sepkowitz et al., 1995; Stuck et al., 1989); b. receiving biological immune modulators (Davis and Ballas, 2017; Winthrop et al., 2018); c. receiving disease-modifying antirheumatic drugs or other immunosuppressive drugs (e.g., cyclosporin, cyclophosphamide, hydroxychloroquine, methotrexate) (Wolfe et al., 2006); (3) pulmonary infection was confirmed by the presence of a new pulmonary infiltrate on chest radiograph, combined with at least two of the following: cough, sputum production, temperature exceeding 38°C or falling below 35°C, auscultatory signs indicative of pneumonia, CRP levels above 15 mg/L, leukocyte counts >10×10^9^/L or <4×10^9^/L, or >10% rods in leucocyte differentiation (Meijvis et al., 2011). Exclusion criteria were as follows: (1) patients who are HIV-Ab positive; (2) patients after radiotherapy or chemotherapy for solid tumors; (3) patients with missing data of OI on the day of admission and on day 7.

### 2.2 Data collection

Data of enrolled patients were extracted from the electronic medical record system, including demographics, underlying diseases, immunosuppressants, laboratory tests at admission, mNGS testing results, CMT results, and clinical outcomes. The laboratory data at admission included white blood cell (WBC), lymphocyte, monocyte, neutrophil, neutrophil-to-lymphocyte ratio (NLR), platelet, C-reactive protein (CRP), lactate dehydrogenase (LDH), D-dimer, procalcitonin (PCT), and oxygenation index (OI). The mNGS test and CMT were conducted within 48 hours of admission.

### 2.3 CMT

CMT was performed on blood, sputum, and bronchoalveolar lavage fluid (BALF) samples collected within 48 hours of admission. The CMT panel included direct smear microscopy, bacterial and fungal cultures, and a cryptococcal antigen test. Targeted PCR was performed for specific pathogens: influenza A/B, SARS-CoV-2, and respiratory syncytial virus (RSV) in sputum or BALF samples, and for cytomegalovirus (CMV) in peripheral blood. Direct smears were used for initial tuberculosis screening and to assess bacterial pathogens.

### 2.4 mNGS of BALF

#### 2.4.1 Sample processing and nucleic acid extraction

BALF samples were collected within 48 hours after admission and immediately transported on dry ice to a commercial laboratory for mNGS testing within 8 hours after collection. BALF samples were liquefied with 0.1% dithiothreitol (DTT) at 56°C for 20 minutes prior to extraction. DNA was then extracted using the TIANamp Magnetic DNA Kit (TIANGEN, China) following the manufacturer’s protocol. The concentration and purity of the extracted DNA were assessed using Qubit 2.0 Fluorometers and Nanodrop 8000 spectrophotometers (Thermo Fisher Scientific, USA), respectively.

#### 2.4.2 Library preparation and quality control

DNA libraries were constructed using the Hieff NGS^®^ C130P2 OnePot II DNA Library Prep Kit for MGI (Yeasen Biotech, China) in accordance with the manufacturer’s instructions. The fragment size distribution and concentration of the constructed libraries were evaluated using the Agilent 2100 Bioanalyzer system (Agilent Technologies, Santa Clara, USA) and the Qubit™ dsDNA HS Assay Kit (Thermo Fisher Scientific, USA). No template controls (NTCs) were included throughout the library preparation process.

#### 2.4.3 Sequencing

Sequencing was performed on the DIFSEQ-200 platform in single-end 50 bp mode, in accordance with the manufacturer’s standard sequencing protocol.

#### 2.4.4 Bioinformatic analysis

Raw sequencing data were demultiplexed and converted to FASTQ format using bcl2fastq2 (version 2.20). Quality filtering was performed with Trimmomatic (version 0.36) to remove low-quality reads, adapter sequences, duplicates, and short reads (<36 bp). Human host sequences were subtracted by alignment to the human reference genome (hs37d5) using Bowtie2 (version 2.2.6). The remaining non-human reads were classified for microbial identification and abundance estimation using Kraken2 (version 2.0.7) and Bracken (version 2.5.0), respectively, against a custom microbial genome database. This database comprised genomes or scaffolds of bacteria, fungi, viruses, and parasites downloaded from GenBank (release 238, http://ftp.ncbi.nlm.nih.gov/genomes/genbank/).

#### 2.4.5 Preliminary interpretation of mNGS results

In the absence of a standardized framework for interpreting mNGS results, we used the following criteria derived from Peng et al (Peng et al., 2021). to define a clinically significant microorganism (CSM):

Bacteria (excluding mycobacteria), fungi (excluding molds), viruses, and parasites: A pathogen was defined as clinically significant if its relative abundance at the species level exceeded 30% and was supported by established literature evidence of pulmonary pathogenicity.

Mycobacterium: Due to its low environmental contamination rate and typically low yield in samples, any species of Mycobacterium with a stringently mapped read number (SMRN) greater than 3 was considered clinically significant.

Molds (including Aspergillus spp., Rhizopus spp., and Mucor spp.): To balance the risk of environmental contamination against the technical challenge of DNA extraction, molds with literature-proven pulmonary pathogenicity were classified as clinically significant when the SMRN at the species level exceeded 10.

Oral commensals were generally excluded from being considered clinically significant, irrespective of their relative abundance, unless there was compelling clinical evidence to the contrary as determined by the managing physician.

#### 2.4.6 Causative pathogen diagnosis of mNGS results

Following the preliminary interpretation of mNGS results, causative pathogen diagnosis in this study was determined by two experienced clinicians based on epidemiology, clinical presentation, treatment outcome, laboratory findings, imaging findings, respiratory pathogenicity of the pathogen, and host immune status. If two clinicians disagree, an in-depth discussion is held with another senior specialist to reach a consensus (Qiu et al., 2025).

### 2.5 Outcomes

The primary outcomes were 7-day OI improvement and clinical improvement up to day 14 based on a 7-category ordinal scale. 7-day OI improvement was defined as a >30% improvement in the OI by day 7 of admission compared to baseline values at the time of hospitalization. The 7-category ordinal scale consisted of the following categories: 1, not hospitalized with resumption of normal activities; 2, not hospitalized, but unable to resume normal activities; 3, hospitalized without supplemental oxygen; 4, hospitalized supplemental oxygen; 5, hospitalized with non-invasive ventilation and high-flow oxygen support; 6, hospitalized with ECMO and/or invasive mechanical ventilation; 7, death (Cao et al., 2020; Wang et al., 2019). Clinical improvement was defined as a decline of two categories on the 7-category ordinal scale.

Secondary outcomes included 21-day mortality and the development of septic shock during hospitalization. The 21-day mortality was defined as death from all causes occurring within 21 days after hospital admission. The diagnosis of septic shock requires both of the following clinical criteria: (1) persistent hypotension necessitating vasopressor therapy to maintain a mean arterial pressure ≥65 mmHg; (2) serum lactate levels >2 mmol/L (18 mg/dL), after adequate volume resuscitation to exclude hypovolemia as the underlying cause (Singer et al., 2016).

### 2.6 Propensity score matching analysis

PSM analysis was performed with IBM SPSS software, version 27.0 (IBM Corp., Armonk, NY, United States), which was used to equalize covariate differences between groups and reduce selection bias. In this study, 7 variables including age, gender, connective tissue diseases, corticosteroids in monotherapy before admission, lymphocyte counts, PCT, and OI < 200 mmHg were matched in a 1:1 non-return match for PSM analysis. A caliper width of 0.05 (on the logit scale of the propensity score) was selected for matching to ensure that matched pairs had similar propensity scores while retaining sufficient sample size for analysis (Austin, 2011). Post-matching balance was assessed using standardized mean differences (SMDs), with a threshold of >0.1 indicating an imbalance between groups.

The results were obtained following PSM, with 82 patients being divided into two groups. We analyzed the impact of BALF mNGS on clinical outcomes in the propensity score-matched cohorts, with results visualized via Sankey diagrams generated using OriginPro (version 2024b; OriginLab Corp., Northampton, MA, USA).

### 2.7 Statistical analysis

Measurement data were tested for normality using the Kolmogorov-Smirnov test or the Shapiro-Wilk test. Continuous variables were expressed as means and standard deviations or medians and interquartile ranges. Categorical variables were presented as frequencies and percentages. Between-group comparisons were performed using independent samples t-tests for normally distributed continuous variables, Wilcoxon rank-sum tests for non-normally distributed continuous variables, and Pearson’s chi-square or Fisher’s exact tests for categorical variables, as appropriate. Absolute risk differences (ARDs) and their 95% confidence intervals were calculated using Newcombe’s method. Two-tailed P<0.05 was considered statistically significant. As this study has four outcomes, Bonferroni correction was performed to account for multiple comparisons across the four pre-specified clinical outcomes, resulting in an adjusted significance threshold of α=0.0125. Kaplan-Meier survival analysis and the log-rank test to determine P values for survival curves were performed with GraphPad Prism Version 10.0 (GraphPad Software, San Diego, CA). Other statistical analyses were performed using IBM SPSS software, version 27.0 (IBM Corp., Armonk, NY, United States).

## 3 Results

### 3.1 Baseline characteristics before matching

**Figure 1:**
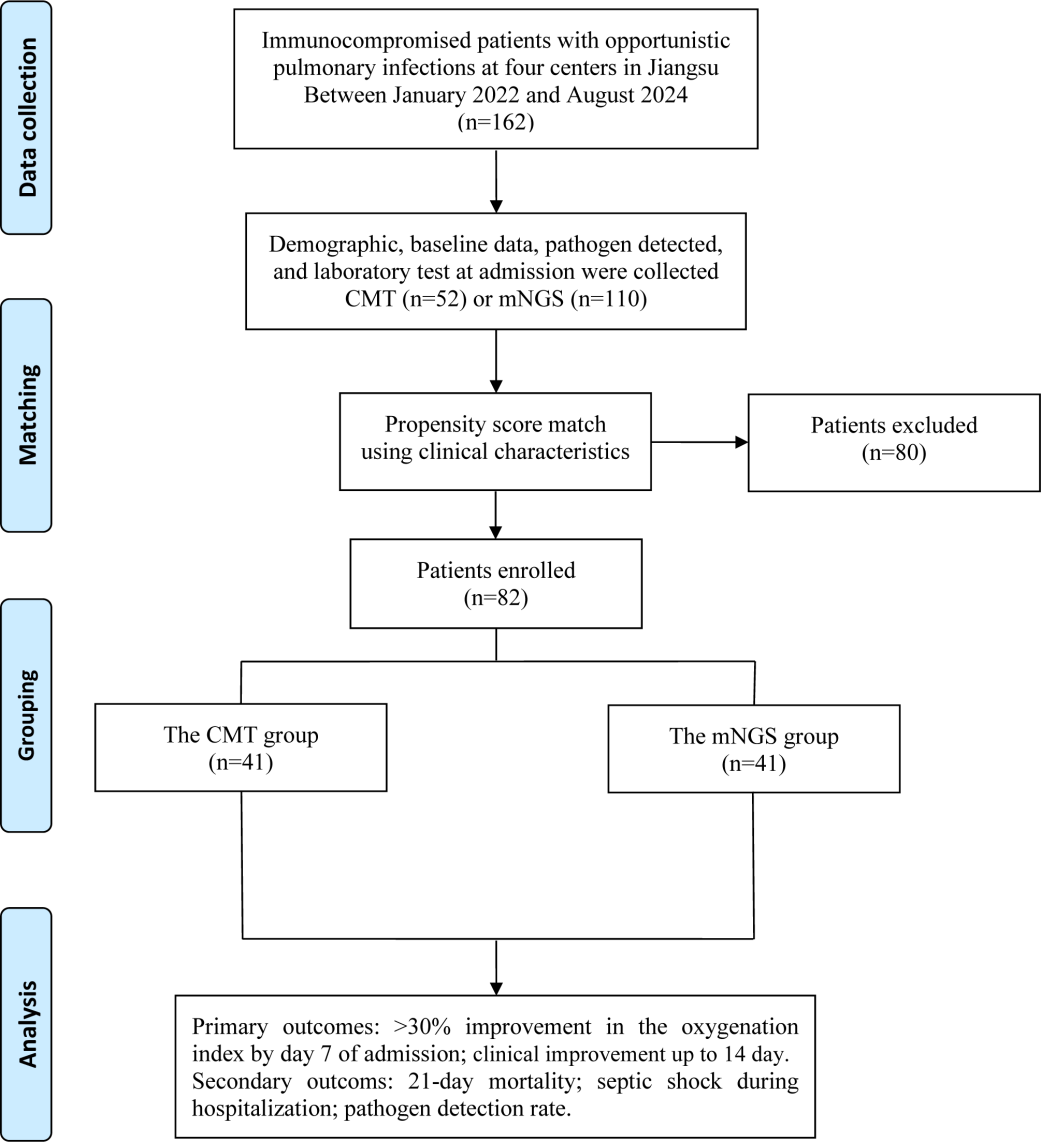
Flow chart of the study.

The study followed a specific flow chart to conduct every step (**Figure 1**). Baseline characteristics of the patients are shown in **Tables 1**, **2**. A total of 162 immunocompromised patients who met the inclusion and exclusion criteria were included in the final analysis. 110 patients underwent the mNGS test and CMT, while 52 patients underwent only CMT following hospital admission.

**Table 1:** Clinical characteristics between the CMT and mNGS groups before and after PSM.

Variables	Unmatched		P value	PSM (1:1)		P value
	CMT	mNGS		CMT	mNGS	
	(n=52)	(n=110)		(n=41)	(n=41)	
Demographics						
Age [y, Mean ± SD]	68.00 (52.50-74.50)	66.00 (54.00-72.00)	0.568	66.00 (51.75-73.75)	68.00 (54.00-72.00)	0.766
Gender [male, n, (%)]	29 (55.8)	59 (53.6)	0.799	25 (61.0)	24 (58.5)	0.822
Underlying diseases [n, (%)]						
Connective tissue diseases	40 (76.9)	57 (51.8)	0.002	29 (70.7)	27 (65.9)	0.635
Nephrotic syndrome	3 (5.8)	12 (10.9)	0.390	3 (7.3)	3 (7.3)	1.000
Solid organ transplantation	2 (3.8)	6 (5.5)	0.659	2 (4.9)	0 (0.0)	0.494
Hematologic malignancies	7 (13.5)	28 (25.5)	0.083	7 (17.1)	7 (17.1)	1.000
Other^a^	0 (0.0)	7 (6.4)	0.067	0 (0.0)	4 (9.8)	0.116
Immunosuppressants [n, (%)]						
Corticosteroids^b^	28 (53.8)	41 (37.3)	0.046	18 (43.9)	19 (46.3)	0.824
Other^c^	5 (9.6)	18 (16.4)	0.251	5 (12.2)	4 (9.8)	0.724
Combination^d^	19 (36.5)	51 (46.4)	0.239	18 (43.9)	18 (43.9)	1.000
Duration [d, M (IQR)]	240.00 (42.50-730.00)	300.00 (60.00-1095.00)	0.594	240.00 (30.00-730.00)	240.00 (42.00-1095.00)	0.858

^a^Other underlying diseases that meet the inclusion criteria. ^b^Corticosteroids in monotherapy. ^c^Immunosuppressive agents other than glucocorticoids. ^d^Corticosteroids in coadministration with other immunosuppressive agents.

**Table 2:** Laboratory characteristics at admission between the CMT and mNGS groups before and after PSM.

Variables	Unmatched		P value	PSM (1:1)		P value
	CMT	mNGS		CMT	mNGS	
	(n=52)	(n=110)		(n=41)	(n=41)	
WBC [×10^9^/L, M (IQR)]	7.70 (5.58-11.89)	7.68 (4.51-11.32)	0.500	8.18 (5.35-12.96)	8.27 (5.36-11.30)	0.673
Lymphocyte [×10^9^/L, M (IQR)]	1.04 (0.59-1.53)	0.62 (0.33-1.16)	0.004	1.04 (0.56-1.77)	0.78 (0.38-1.30)	0.268
Monocyte [×10^9^/L, M (IQR)]	0.46 (0.25-0.69)	0.35 (0.14-0.57)	0.127	0.46 (0.25-0.72)	0.42 (0.22-0.67)	0.981
Neutrophil [×10^9^/L, M (IQR)]	5.99 (4.32-9.87)	5.98 (3.45-10.45)	0.747	6.93 (4.28-10.52)	6.82 (3.99-9.85)	0.578
NLR [M (IQR)]	6.54 (3.73-14.70)	10.07 (4.42-27.00)	0.055	6.57 (3.62-16.08)	11.43 (3.82-19.40)	0.180
PLT [×10^9^/L, Mean ± SD]	191.00 (115.50-240.00)	172.00 (108.00-242.00)	0.732	192.00 (107.75-242.50)	198.00 (128.00-265.00)	0.718
CRP [mg/L, M (IQR)]	29.09 (8.82-118.78)	56.05 (11.25-137.47)	0.159	51.96 (11.97-122.52)	21.17 (10.77-93.10)	0.970
LDH [U/L, M (IQR)]	304.95 (223.00-411.25)	306.90 (238.30-435.60)	0.610	304.95 (220.15-390.83)	260.70 (194.00-383.90)	0.137
D-d [μg/ml, M (IQR)]	1.27 (0.51-2.18)	1.29 (0.51-2.98)	0.582	1.18 (0.50-2.96)	1.46 (0.42-4.16)	0.444
PCT [ng/ml, M (IQR)]	0.13 (0.08-0.14)	0.14 (0.08-0.55)	0.020	0.12 (0.06-0.14)	0.13 (0.07-0.29)	0.346
OI [mmHg, M (IQR)]	389.00 (223.07-476.19)	235.00 (147.37-335.00)	<0.001	340.50 (221.00-475.89)	293.30 (218.00-356.00)	0.447
<200mmHg [n, (%)]	9 (17.3)	41 (37.3)	0.010	9 (22.0)	7 (17.1)	0.577
≥200mmHg [n, (%)]	43 (82.7)	69 (62.7)	0.010	32 (78.0)	34 (82.9)	0.577

CMT, conventional microbiological tests; mNGS, metagenomic next-generation sequencing; M (IQR), median (Interquartile Range); WBC, white blood cell; NLR, neutrophil-to-lymphocyte ratio; PLT, platelet; CRP, C-reactive protein; LDH, lactate dehydrogenase; D-d, D-dimer; PCT, procalcitonin; OI, oxygenation index.

Before PSM, the CMT and mNGS groups exhibited significant differences in baseline characteristics. Connective tissue diseases were the most common underlying conditions, including systemic lupus erythematosus (SLE, n=14), rheumatoid arthritis (RA, n=15), Sjogren’s syndrome (SS, n=3), systemic sclerosis (SSc, n=1), polymyositis (PM, n=1), dermatomyositis (DM, n=15), mixed connective tissue disease (MTCD, n=33), and systemic vasculitis (n=15). The CMT group had a higher prevalence of connective tissue diseases (76.9% vs. 51.8%, P = 0.002) and more frequent corticosteroid monotherapy prior to admission (53.8% vs. 37.3%, P = 0.046; **Table 1**). Additionally, the mNGS group showed higher PCT levels (0.14 vs. 0.13 ng/mL, P = 0.020), along with lower lymphocyte counts (0.62 vs. 1.04×10^9^/L, P = 0.004) and OI (235 vs. 389 mmHg, P<0.001) at admission (**Table 2**). These imbalances necessitated PSM to minimize confounding effects in subsequent analyses.

### 3.2 Baseline characteristics were matched via PSM analysis

**Figure 2:**
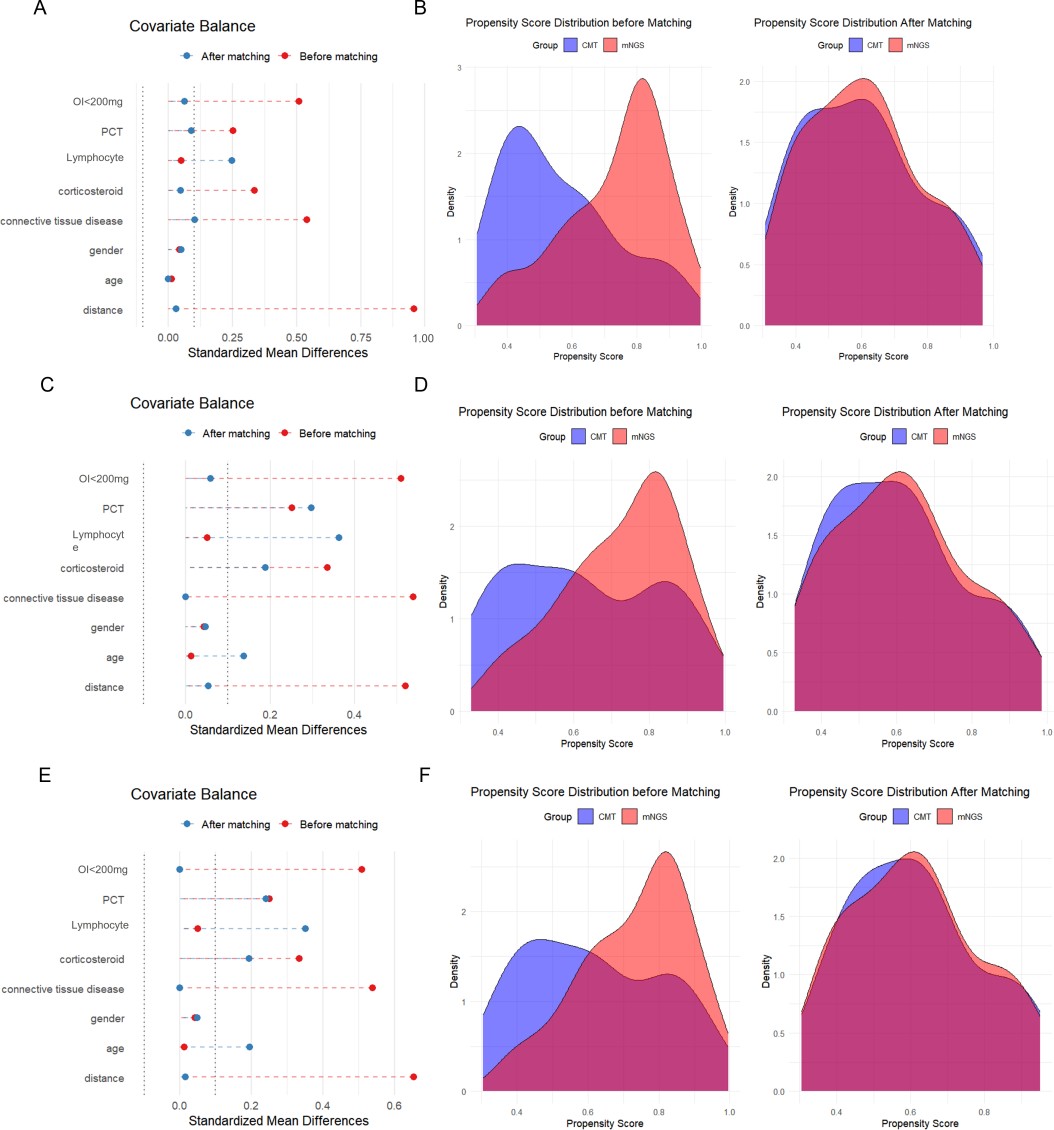
Evaluation of propensity score matching. **(A)** Covariate balance before and after matching with a caliper width of 0.05. **(B)** Distribution of propensity scores with a caliper width of 0.05. **(C)** Covariate balance before and after matching with a caliper width of 0.04. **(D)** Distribution of propensity scores with a caliper width of 0.04. **(E)** Covariate balance before and after matching with a caliper width of 0.03. **(F)** Distribution of propensity scores with a caliper width of 0.03.

Following 1:1 propensity score matching (PSM), the study cohort comprised 41 patients from each group (CMT and mNGS), with all baseline covariates demonstrating adequate balance (all P>0.05). As shown in **Figures 2A, B**, the standardized mean differences of the covariates decreased after matching and fell below the recommended threshold of 0.1, indicating that the cohort achieved a good balance. Furthermore, the density plots of the propensity score distributions revealed a markedly greater overlap between the two groups after matching compared with that before matching. The above results confirmed that PSM successfully generated comparable groups, thereby enabling a more robust evaluation of BALF-mNGS effects.

After PSM, clinical characteristics including age, gender, underlying diseases, and immunosuppressant usage demonstrated no statistically significant differences (**Table 1**). **Table 2** presents the comparison of laboratory characteristics at admission between the two groups after PSM. It was indicated that hematological indices, inflammatory markers, tissue injury biomarkers, and OI were comparable. Subsequent analyses focused on assessing the clinical impact of BALF mNGS in this matched cohort.

### 3.3 Primary outcomes

**Figure 3:**
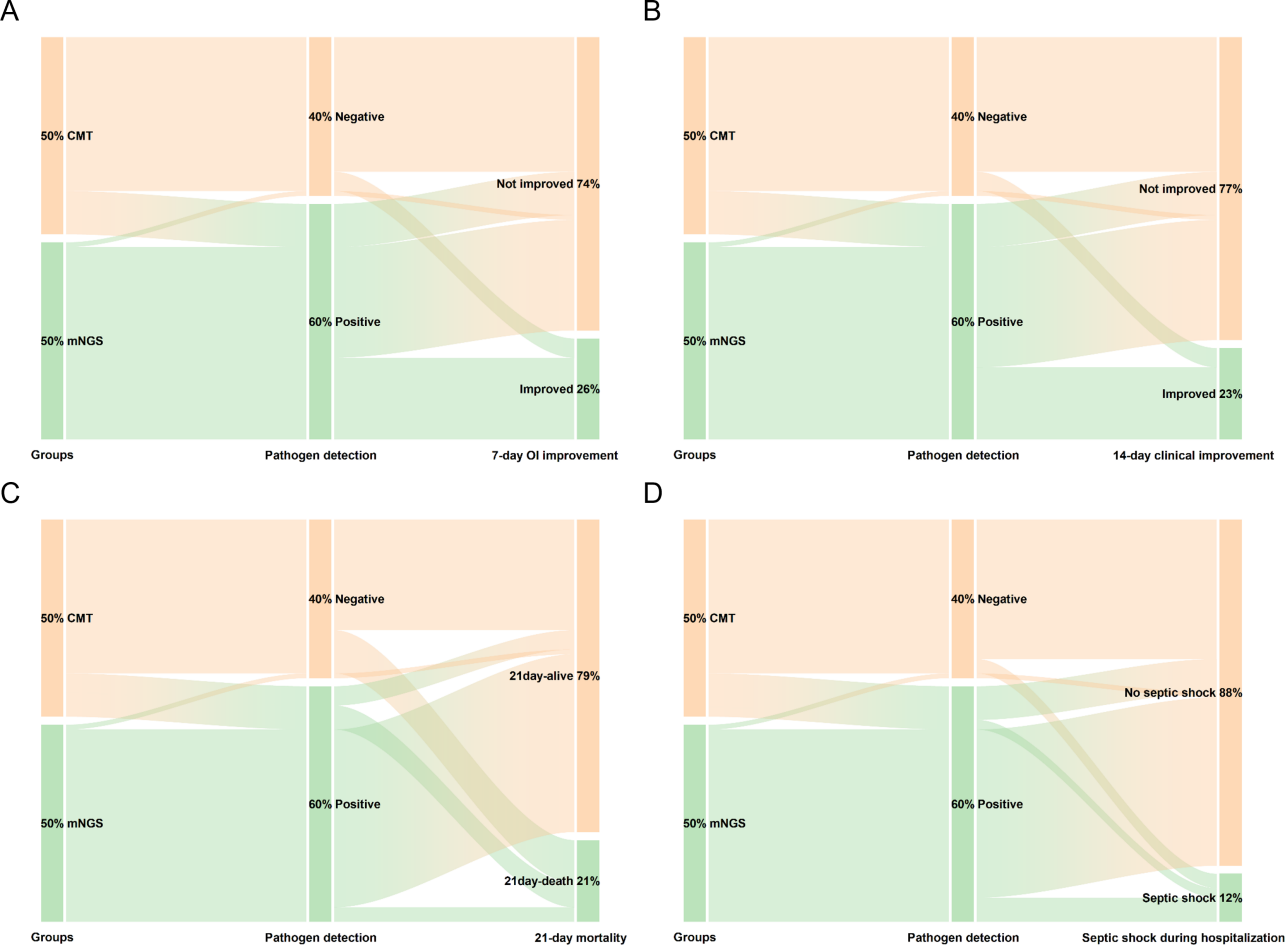
This Sankey diagram visually demonstrates that utilizing mNGS (versus CMT) significantly increases pathogen detection rates, thereby guiding a greater proportion of patients toward clinical improvement and survival. **(A)** 7-day OI improvement: A significantly higher proportion of patients in the mNGS group achieved this improvement compared to the CMT group (41.5% vs. 9.8%; P = 0.001); **(B)** 14-day clinical improvement: The mNGS group showed a markedly higher rate of clinical improvement by day 14 (36.6% vs. 9.8%; P = 0.004); **(C)** 21-day mortality: The mNGS group had significantly lower all-cause mortality within 21 days of admission compared to the CMT group (7.3% vs. 34.1%; P = 0.003); **(D)** Septic shock during hospitalization: The incidence of septic shock was comparable between the two groups (12.2% vs. 12.2%; P = 1.000), indicating no significant difference in this secondary outcome.

Clinical outcomes in both groups are presented in **Figure 3** and **Table 3**. Firstly, OI was assessed at admission and on day 7 post-admission. Compared to the CMT group, a significantly greater proportion of patients in the mNGS group achieved >30% improvement in OI by day 7 (41.5% vs. 9.8%; P = 0.001; absolute risk difference [ARD]=31.7%, 95% CI: 13.0% to 48.0%). Additionally, we evaluated 14-day clinical improvement using the WHO 7-category ordinal scale as another primary outcome. Consistent with the oxygenation improvement findings, the mNGS group exhibited higher rates of clinical improvement by day 14 than the CMT group (36.6% vs. 9.8%; P = 0.004; ARD = 26.8%, 95% CI: 8.6% to 43.2%).

**Table 3:** Main clinical outcomes of the study after PSM.

Variables	CMT	mNGS	P value	Absolute risk difference
	(n=41)	(n=41)		(95% CI)
The primary clinical outcome				
7-day OI improvement^a^	4 (9.8%)	17 (41.5%)	0.001	31.7% (13.0% to 48.0%)
14-day clinical improvement^b^	4 (9.8%)	15 (36.6%)	0.004	26.8% (8.6% to 43.2%)
Secondary clinical outcomes				
21-day mortality	14 (34.1%)	3 (7.3%)	0.003	−26.8% (−42.9% to −9.4%)
Septic shock development	5 (12.2%)	5 (12.2%)	1.000	0.0% (−15.0% to 15.0%)
Pathogen detection rate	9 (22.0%)	40 (97.6%)	<0.001	75.6% (63.5% to 87.7%)

^a^7-day OI improvement was defined as a >30% improvement in the oxygenation index (OI) by day 7 of admission compared to baseline values at the time of hospitalization.

^b^14-day clinical improvement was defined as a decrease of 2 categories on a 7-category ordinal scale up to day 14 after admission.

CMT, conventional microbiological tests; mNGS, metagenomic next-generation sequencing.

### 3.4 Secondary outcomes

**Figure 4:**
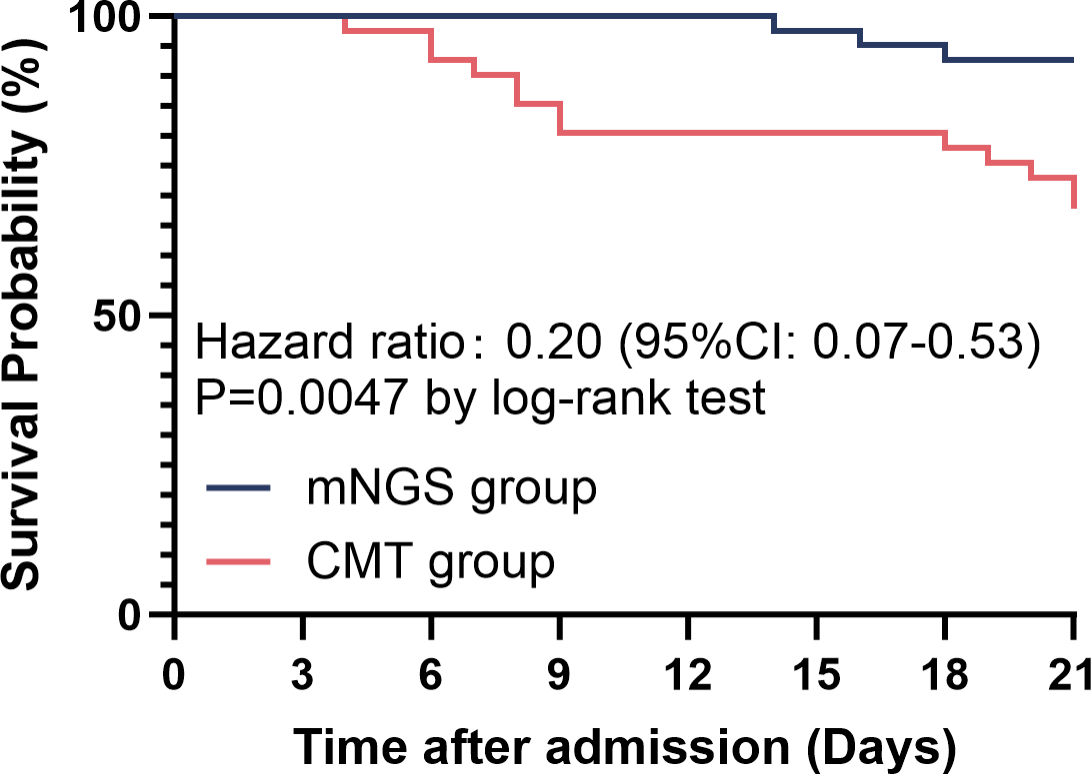
Kaplan-Meier survival analysis comparing 21-day survival rates between CMT and mNGS groups after PSM. The 21-day survival was significantly higher in the mNGS group than in the CMT group (HR: 0.20, 95%CI: 0.07-0.53, P = 0.005).

In the enrolled subjects, the application of BALF mNGS was significantly associated with reduced 21-day mortality compared to CMT (7.3% vs. 34.1%, P = 0.003; ARD=−26.8%, 95% CI: −42.9% to −9.4%). This mortality benefit was corroborated by Kaplan-Meier survival analysis (**Figure 4**), highlighting the positive clinical impact of mNGS-guided therapeutic decisions. However, despite the survival advantage, the incidence of septic shock during hospitalization did not differ significantly between groups (P>0.05). Totally, the mNGS group showed a significantly higher overall pathogen detection rate compared to CMT (97.6% vs. 22.0%, P<0.001; ARD = 75.6%, 95% CI: 63.5% to 87.7%; **Table 3**).

### 3.5 Sensitivity analysis to verify the robustness of study endpoints

To verify the robustness of our primary propensity score matching, we conducted sensitivity analyses by varying the caliper width to 0.04 and 0.03. As demonstrated in **Figures 2C–F**, both alternative matching strategies achieved excellent covariate balance between the CMT and mNGS groups, with the SMD of propensity score falling below the 0.1 threshold after matching. Propensity score distribution plots further confirmed it.

Given the assessment of primary and secondary outcomes, we performed a sensitivity analysis using the Bonferroni method to control the family-wise error rate. The significance threshold was adjusted to α=0.0125 (0.05/4). After this correction, the between-group differences in 7-day OI improvement (P = 0.001), 14-day clinical improvement (P = 0.004), and 21-day mortality (P = 0.003) remained statistically significant. The result for septic shock incidence remained non-significant.

### 3.6 mNGS demonstrated a higher diagnostic yield compared to CMT

**Figure 5:**
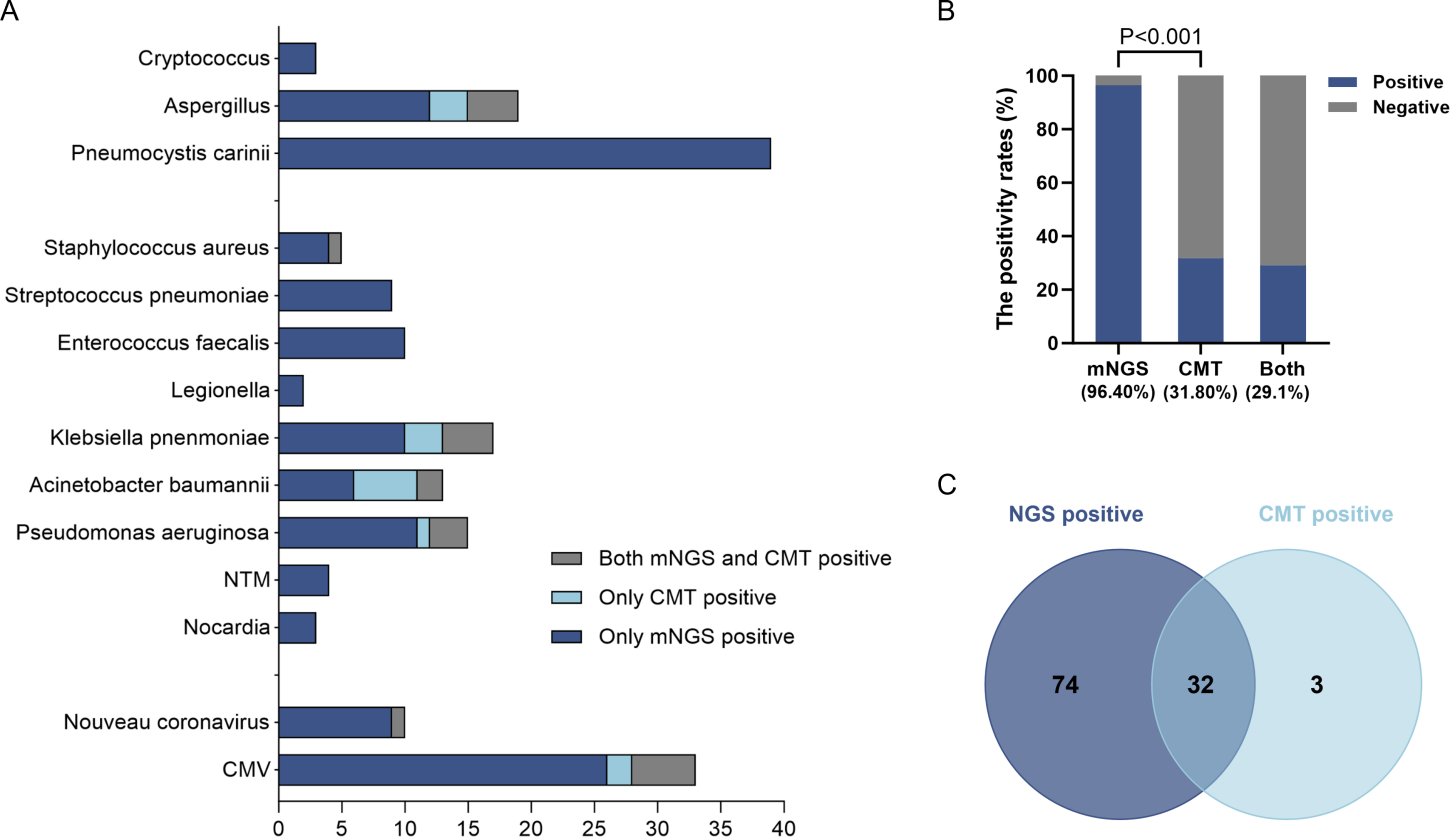
Pathogens detected by mNGS and CMT in the mNGS group. **(A)** Distribution of pathogens identified by mNGS and CMT; **(B)** Comparative detection rates of mNGS, CMT, and their combination (96.40% vs. 31.80% vs. 14.50%); **(C)** Concordance between results of mNGS and CMT who receiving combined examination at admission (22/35).

We assessed the concordance between mNGS and CMT results for the 110 patients who underwent both tests at admission. Detailed pathogens of each patient are presented in **Supplementary Tables S1, S2**. As shown in **Table 4** and **Figure 5**, mNGS detected causative pathogens in 96.4% of cases (106/110), significantly higher than the positivity rate of CMT (31.8%, 35/110). Among the 35 CMT-positive cases, 32 (91.4%) were also positive by mNGS, indicating partial concordance between the two methods. Only 4 cases (3.6%) were negative by both methods. The considerable difference in positivity rates suggests that mNGS may detect pathogens missed by conventional methods.

**Table 4:** Comparison of CMT results and mNGS results in the group who receiving combined examination at admission.

Microbiological findings	CMT (n=110)	mNGS (n=110)	P value
Only virus [n, (%)]	2 (1.8)	8 (7.3)	0.052
Only bacteria [n, (%)]	10 (9.1)	11 (10.0)	0.819
Only fungi excluding PJ [n, (%)]	16 (14.5)	9 (8.2)	0.137
PJ [n, (%)]	0 (0.0)	16 (14.5)	<0.001
Co-infection [n, (%)]	9 (8.2)	64 (58.2)	<0.001
Bacteria + CMV	5 (4.5)	13 (11.8)	0.049
Bacteria + PJ	0 (0.0)	11 (10.0)	<0.001
Bacteria + Aspergillus	1 (0.9)	3 (2.7)	0.313
PJ + CMV	0 (0.0)	12 (10.9)	<0.001
PJ + Aspergillus	0 (0.0)	3 (2.7)	0.247
CMV + Aspergillus	1 (0.9)	5 (4.5)	0.212
Overall positivity [n, (%)]	35 (31.8)	106 (96.4)	<0.001

CMT, conventional microbiological tests; mNGS, metagenomic next-generation sequencing; PJ, Pneumocystis jiroveci.

The diagnostic superiority of mNGS was particularly evident in two key aspects. First, it identified several challenging respiratory pathogens that were undetectable by CMT, including *Nocardia* (2.7% vs. 0.0%), non-tuberculous mycobacteria (NTM, 3.6% vs. 0.0%), *Legionella* (1.8% vs. 0.0%), PJ (35.5% vs. 0.0%), CMV (28.2% vs. 6.4%), and *Cryptococcus* (2.7% vs. 0.0%). Second, the detection rate of co-infection using mNGS was significantly higher than that of CMT (58.2% vs. 8.2%; P<0.001). Bacteria and CMV co-infection represented the most prevalent combination in this study (18/110, 16.4%).

### 3.7 Antibiotic therapy adjustment according to mNGS results

The impact of BALF-mNGS results on antibiotic therapy was further analyzed in the 110 patients who underwent both mNGS and CMT at admission (**Table 5**). Based on the interpreted mNGS results, clinicians promptly adjusted antibiotic therapy for patients who required it. In this study, antibiotic therapy was modified in 89 patients. In detail, antimicrobial coverage was broadened for 55 patients (50.0%) and narrowed for 12 patients (10.9%). For 22 patients (20.0%), mNGS prompted both an expansion of coverage for newly identified pathogens and a de-escalation for organisms that were not detected. No change to the antibiotic regimen was made for the remaining 21 patients (19.1%) following mNGS testing.

**Table 5:** Impact of mNGS results on antibiotic stewardship.

Variables	Population (n=110)
Any modification [n, (%)]	89 (80.9)
Increase coverage only [n, (%)]	55 (50.0)
Decrease coverage only [n, (%)]	12 (10.9)
Both increase and decrease [n, (%)]	22 (20.0)
No modification [n, (%)]	21 (19.1)

## 4 Discussion

This real-world study focused on the application of BALF mNGS in immunocompromised patients with opportunistic pulmonary infections, which suggested that BALF mNGS was associated with improved OI up to day 7, and enhanced clinical improvement up to day 14. Moreover, the study confirmed that mNGS contributed to a reduction in 21-day mortality, likely attributed to its higher pathogen detection rate and accelerated diagnostic time for pulmonary infections.

In immunocompetent hosts, a study in which we participated showed that mNGS significantly improved clinical outcomes in patients with severe community-acquired pneumonia (Wu et al., 2025). The present study further confirmed that mNGS demonstrates superior diagnostic performance over CMT for lower respiratory tract infections in immunocompromised patients. This advantage is particularly evident in detecting pathogens like PJ, which increasingly causes infections in non-HIV immunocompromised hosts. BALF mNGS and real-time PCR are recognized as highly sensitive and specific methods for the rapid diagnosis of *Pneumocystis jirovecii* pneumonia (PJP) (Chen et al., 2024). However, PCR for *Pneumocystis jirovecii* was not yet widely scaled or available in the four respiratory centers, which may have some impact on pathogen coverage in the CMT group. Furthermore, the diagnostic value of *Pneumocystis jiroveci* PCR is limited in identifying mixed infections. Since mixed infections often occur in opportunistic pulmonary infections in immunosuppressed hosts, clinicians tend to choose mNGS more often for the patient’s condition, which is more conducive to appropriate and timely treatment. This study indicated a high rate of co-infections, particularly between bacteria and CMV, which is associated with increased mortality and presents significant management challenges in immunocompromised critically ill patients (Fernández et al., 2025). Therefore, the simultaneous detection of both pathogens by mNGS is critical, as it provides a rationale for initiating or broadening antimicrobial coverage while simultaneously instituting targeted anti-viral therapy. However, the cost of mNGS should be taken into consideration in clinical practice. Therefore, for immunocompetent patients with common infections, CMT remains the preferred choice. However, for high-risk populations such as immunosuppressed hosts with opportunistic pulmonary infections, the diagnostic efficacy of mNGS enables early initiation of precise antibiotic therapy, reduces empirical treatment, and shortens hospital stays, thereby indirectly lowering the overall medical costs. The supplementary tables further provide the pathogen landscape identified by mNGS in our cohort, which revealed not only the high prevalence of expected opportunistic pathogens like PJ and cytomegalovirus but also highlighted the detection of fastidious, atypical, or rarely cultured organisms. Notable examples included Nocardia, Legionella, NTM, and Cryptococcus, each of which required distinct antimicrobial regimens fundamentally different from empirical broad-spectrum antibacterial therapy (Diao et al., 2021). The timely identification of these pathogens can help clinicians initiate targeted antimicrobial therapy earlier, which may have contributed to the observed improvements in oxygenation and survival.

While mNGS has been previously shown to enhance treatment strategies and increase diagnostic yield in immunocompromised patients with pneumonia (Azar et al., 2021; Sun et al., 2021; Tang et al., 2021), studies regarding its clinical impact are scarce. Furthermore, existing evidence suggested that mNGS contributes to better clinical outcomes, including reduced ICU length of stay, shorter duration of mechanical ventilation, lower ICU hospitalization costs, and decreased 60-day mortality in this vulnerable population (Zhang et al., 2022, 2020). However, it was unclear whether the mNGS benefits the OI improvement, clinical improvement, and incidence of septic shock during hospitalization. Therefore, this study assessed the clinical utility of mNGS in immunocompromised hosts with opportunistic pulmonary infections to demonstrate its potential to improve early management and clinical outcomes. To our knowledge, it is the first to assess the impact of BALF-mNGS on early clinical outcomes in this specific population.

The improvement in oxygenation in the mNGS group is clinically significant. Previously, Rosa et al. indicated that oxygenation improvement was associated with ICU mortality in mechanically ventilated COVID-19 patients (De Rosa et al., 2025). In this study, the mNGS group showed a significantly greater proportion of patients achieving >30% improvement in OI by day 7 compared to the CMT group, suggesting a potential clinical benefit of mNGS in early respiratory recovery. Importantly, as failure to achieve early OI improvement has been validated as a predictor of therapeutic failure in acute respiratory distress syndromes (Go et al., 2016), our results suggested that the application of BALF mNGS could potentially modify disease regression in severe respiratory infections.

The 7-category ordinal scale was used to assess the clinical improvement up to day 14, which was more frequent in the mNGS group than the CMT group in this study. The mNGS offers broader pathogen spectrum coverage, demonstrating significant advantages over conventional detection methods (Gao et al., 2024). Furthermore, its ability to provide rapid and accurate pathogenic diagnosis facilitates earlier transition from empiric to targeted therapy, thereby prompting antibiotic treatment adjustment rates and reducing unnecessary antibiotic use (Li et al., 2023; Wu et al., 2025), which suggests that mNGS is critical to clinical decision making. Immunocompromised hosts are more susceptible to mixed infections compared to immunocompetent individuals (Kreitmann et al., 2024). CMT in this population often fails to detect all causative pathogens, leading to high false-negative rates (Duan et al., 2021). Consequently, the value of mNGS in immunocompromised patients with opportunistic pulmonary infections is more prominent, which probably accounts for significantly higher clinical improvement rates on day 14 after admission compared to CMT.

The present study first showed that mNGS was significantly associated with 21-day mortality in immunocompromised patients with opportunistic pulmonary infections. Pulmonary infections pose a substantial global health threat for immunocompromised patients, in whom delayed diagnosis and empiric antibiotic therapy often lead to adverse outcomes (Wang et al., 2023). It has been illustrated that a delay in diagnosis is associated with mortality in immunocompromised patients with pulmonary infections. Li et al (Li et al., 2014). concluded that a delay in the initiation of antimicrobial therapy for PJP was more common in the non-HIV-infected patients and was most likely related to the poor prognosis. Compared to traditional culture, mNGS offers a clinically significant reduction in time to diagnosis. This is especially critical when managing opportunistic pulmonary infections in immunocompromised patients, who often present with a wide range of possible pathogens that are difficult to anticipate empirically. In clinical practice, relying solely on targeted PCR that requires pre-suspicion of specific pathogens increases the likelihood of missing atypical or co-infecting organisms. With its untargeted approach, mNGS provides broad pathogen coverage in a single workflow, which may facilitate timely and evidence-based adjustments to antibiotic therapy. Additionally, CMT may significantly reduce pathogen detection after antibiotic exposure, with a long time to obtain culture results. In contrast, mNGS is less affected by prior antibiotic exposure and significantly faster than that of pathogen cultures (Miao et al., 2018). For immunocompromised patients with pulmonary infections, empirical antibiotic therapy is frequently initiated before pathogen identification. The detection of mNGS enables rapid and precise identification of pathogenic microorganisms, thereby facilitating timely clinical diagnosis and antibiotic adjustments. Such pathogen-targeted therapy may significantly improve clinical management and patient prognosis in this vulnerable population.

This study has several limitations. First, targeted PCR for Pneumocystis was unavailable in the CMT group despite being a common infection, which may lead to bias. However, PCR for Pneumocystis was not widely available or standardized (Zhou et al., 2021). Second, our cohort exclusively comprised immunocompromised patients, and the lack of an immunocompetent control group limits our ability to determine whether the observed benefits of mNGS in this study are unique to this population. Third, a technical limitation of our study is that the mNGS protocol targeted only DNA. RNA viruses, including influenza, SARS-CoV-2, and RSV, are primarily detected via PCR methods; non-pneumonia-causing RNA viruses are not within the detection scope. Fourth, despite the multicenter design, the relatively small sample size and lack of blind design may limit the statistical power and generalizability of our findings. Although we employed PSM to minimize the selection bias, unobserved confounders from unmeasured variables may persist. Additionally, the long-term efficacy after 21 days of mNGS in the immunocompromised patients will also be necessary to be studied in the future.

## 5 Conclusion

In conclusion, early BALF mNGS testing was associated with improved clinical outcomes, including OI improvement at day 7, clinical status recovery, and decreased 21-day mortality, likely facilitated by its higher diagnostic yield and potential for earlier targeted therapy. Further prospective study with a large sample size is needed to better understand the prognostic value of mNGS in this population.

## Data Availability

The raw data supporting the conclusions of this article will be made available by the authors, without undue reservation.

## References

[B1] Austin P. C. (2011). Optimal caliper widths for propensity-score matching when estimating differences in means and differences in proportions in observational studies. Pharm. Stat. 10, 150–161. doi: 10.1002/pst.433, PMID: 20925139 PMC3120982

[B2] Azar M. M. Schlaberg R. Malinis M. F. Bermejo S. Schwarz T. Xie H. . (2021). Added diagnostic utility of clinical metagenomics for the diagnosis of pneumonia in immunocompromised adults. Chest 159, 1356–1371. doi: 10.1016/j.chest.2020.11.008, PMID: 33217418

[B3] Azoulay E. Mokart D. Kouatchet A. Demoule A. Lemiale V. (2019). Acute respiratory failure in immunocompromised adults. Lancet Respir. Med. 7, 173–186. doi: 10.1016/S2213-2600(18)30345-X, PMID: 30529232 PMC7185453

[B4] Azoulay E. Russell L. Van de Louw A. Metaxa V. Bauer P. Povoa P. . (2020). Diagnosis of severe respiratory infections in immunocompromised patients. Intensive Care Med. 46, 298–314. doi: 10.1007/s00134-019-05906-5, PMID: 32034433 PMC7080052

[B5] Cao B. Wang Y. Wen D. Liu W. Wang J. Fan G. . (2020). A trial of lopinavir-ritonavir in adults hospitalized with severe covid-19. N Engl. J. Med. 382, 1787–1799. doi: 10.1056/NEJMoa2001282, PMID: 32187464 PMC7121492

[B6] Chen Q. Chen X. Mo P. Chen L. Du Q. Hu W. . (2024). Diagnostic values of BALF metagenomic next-generation sequencing, BALF real-time PCR and serum BDG for Pneumocystis jirovecii pneumonia in HIV-infected patients. Front. Microbiol. 15, 1421660. doi: 10.3389/fmicb.2024.1421660, PMID: 39372273 PMC11449763

[B7] Chen S. Kang Y. Li D. Li Z. (2022). Diagnostic performance of metagenomic next-generation sequencing for the detection of pathogens in bronchoalveolar lavage fluid in patients with pulmonary infections: Systematic review and meta-analysis. Int. J. Infect. Dis. 122, 867–873. doi: 10.1016/j.ijid.2022.07.054, PMID: 35907477

[B8] Davis B. P. Ballas Z. K. (2017). Biologic response modifiers: Indications, implications, and insights. J. Allergy Clin. Immunol. 139, 1445–1456. doi: 10.1016/j.jaci.2017.02.013, PMID: 28263774

[B9] De Rosa S. Sella N. Bellani G. Foti G. Cortegiani A. Lorenzoni G. . (2025). Oxygenation improvement and duration of prone positioning are associated with ICU mortality in mechanically ventilated COVID-19 patients. Ann. Intensive Care 15, 20. doi: 10.1186/s13613-025-01438-y, PMID: 39875763 PMC11775368

[B10] Diao Z. Han D. Zhang R. Li J. (2021). Metagenomics next-generation sequencing tests take the stage in the diagnosis of lower respiratory tract infections. J. Adv. Res. 38, 201–212. doi: 10.1016/j.jare.2021.09.012, PMID: 35572406 PMC9091713

[B11] Diao Z. Han D. Zhang R. Li J. (2022). Metagenomics next-generation sequencing tests take the stage in the diagnosis of lower respiratory tract infections. J. Adv. Res. 38, 201–212. doi: 10.1016/j.jare.2021.09.012, PMID: 35572406 PMC9091713

[B12] Duan H. Li X. Mei A. Li P. Liu Y. Li X. . (2021). The diagnostic value of metagenomic next⁃generation sequencing in infectious diseases. BMC Infect. Dis. 21, 62. doi: 10.1186/s12879-020-05746-5, PMID: 33435894 PMC7805029

[B13] Fernández S. Castro P. Azoulay E. (2025). What intensivists need to know about cytomegalovirus infection in immunocompromised ICU patients. Intensive Care Med. 51, 39–61. doi: 10.1007/s00134-024-07737-5, PMID: 39774866

[B14] Gao Q. Li L. Su T. Liu J. Chen L. Yi Y. . (2024). A single-center, retrospective study of hospitalized patients with lower respiratory tract infections: clinical assessment of metagenomic next-generation sequencing and identification of risk factors in patients. Respir. Res. 25, 250. doi: 10.1186/s12931-024-02887-y, PMID: 38902783 PMC11191188

[B15] Go L. Budinger G. R. S. Kwasny M. J. Peng J. Forel J.-M. Papazian L. . (2016). Failure to improve the oxygenation index is a useful predictor of therapy failure in acute respiratory distress syndrome clinical trials. Crit. Care Med. 44, e40–ee4. doi: 10.1097/CCM.0000000000001295, PMID: 26427588 PMC5224531

[B16] Kreitmann L. Helms J. Martin-Loeches I. Salluh J. Poulakou G. Pène F. . (2024). ICU-acquired infections in immunocompromised patients. Intensive Care Med. 50, 332–349. doi: 10.1007/s00134-023-07295-2, PMID: 38197931

[B17] Kunihiro Y. Tanaka N. Kawano R. Yujiri T. Kubo M. Ueda K. . (2019). Differential diagnosis of pulmonary infections in immunocompromised patients using high-resolution computed tomography. Eur. Radiol. 29, 6089–6099. doi: 10.1007/s00330-019-06235-3, PMID: 31062135

[B18] Li M.-C. Lee N.-Y. Lee C.-C. Lee H.-C. Chang C.-M. Ko W.-C. (2014). Pneumocystis jiroveci pneumonia in immunocompromised patients: delayed diagnosis and poor outcomes in non-HIV-infected individuals. J. Microbiol. Immunol. Infect. 47, 42–47. doi: 10.1016/j.jmii.2012.08.024, PMID: 23063081

[B19] Li X.-X. Niu C.-Z. Zhao Y.-C. Fu G.-W. Zhao H. Huang M.-J. . (2023). Clinical application of metagenomic next-generation sequencing in non-immunocompromised patients with severe pneumonia supported by veno-venous extracorporeal membrane oxygenation. Front. Cell Infect. Microbiol. 13, 1269853. doi: 10.3389/fcimb.2023.1269853, PMID: 37900317 PMC10613042

[B20] Liu Y. Ma Y. (2024). Clinical applications of metagenomics next-generation sequencing in infectious diseases. J. Zhejiang Univ Sci. B 25, 471–484. doi: 10.1631/jzus.B2300029, PMID: 38910493 PMC11199093

[B21] Liu Y. Wu W. Xiao Y. Zou H. Hao S. Jiang Y. (2024). Application of metagenomic next-generation sequencing and targeted metagenomic next-generation sequencing in diagnosing pulmonary infections in immunocompetent and immunocompromised patients. Front. Cell Infect. Microbiol. 14, 1439472. doi: 10.3389/fcimb.2024.1439472, PMID: 39165919 PMC11333343

[B22] Meijvis S. C. A. Hardeman H. Remmelts H. H. F. Heijligenberg R. Rijkers G. T. van Velzen-Blad H. . (2011). Dexamethasone and length of hospital stay in patients with community-acquired pneumonia: a randomised, double-blind, placebo-controlled trial. Lancet 377, 2023–2030. doi: 10.1016/S0140-6736(11)60607-7, PMID: 21636122

[B23] Miao Q. Ma Y. Wang Q. Pan J. Zhang Y. Jin W. . (2018). Microbiological diagnostic performance of metagenomic next-generation sequencing when applied to clinical practice. Clin. Infect. Dis. 67, S231–SS40. doi: 10.1093/cid/ciy693, PMID: 30423048

[B24] Peng J.-M. Du B. Qin H.-Y. Wang Q. Shi Y. (2021). Metagenomic next-generation sequencing for the diagnosis of suspected pneumonia in immunocompromised patients. J. Infect. 82, 22–27. doi: 10.1016/j.jinf.2021.01.029, PMID: 33609588

[B25] Qian Y.-Y. Wang H.-Y. Zhou Y. Zhang H.-C. Zhu Y.-M. Zhou X. . (2020). Improving pulmonary infection diagnosis with metagenomic next generation sequencing. Front. Cell Infect. Microbiol. 10, 567615. doi: 10.3389/fcimb.2020.567615, PMID: 33585263 PMC7874146

[B26] Qiu D. Wang Y. Guo S. Xue T. Wang A. Chen S. . (2025). Lung puncture biopsies based targeted NGS increase clinical benefit to patients with short-term progressive pulmonary lesions suspected to be benign. Int. J. Infect. Dis. 163, 108232. doi: 10.1016/j.ijid.2025.108232, PMID: 41265634

[B27] Sepkowitz K. A. Brown A. E. Armstrong D. (1995). Pneumocystis carinii pneumonia without acquired immunodeficiency syndrome. More patients same risk. Arch. Intern. Med. 155, 1125–1128. doi: 10.1001/archinte.1995.00430110015002 7763117

[B28] Shahani L. Ariza-Heredia E. J. Chemaly R. F. (2017). Antiviral therapy for respiratory viral infections in immunocompromised patients. Expert Rev. Anti Infect. Ther. 15, 401–415. doi: 10.1080/14787210.2017.1279970, PMID: 28067078 PMC7103713

[B29] Singer M. Deutschman C. S. Seymour C. W. Shankar-Hari M. Annane D. Bauer M. . (2016). The third international consensus definitions for sepsis and septic shock (Sepsis-3). JAMA 315, 801–810. doi: 10.1001/jama.2016.0287, PMID: 26903338 PMC4968574

[B30] Stuck A. E. Minder C. E. Frey F. J. (1989). Risk of infectious complications in patients taking glucocorticosteroids. Rev. Infect. Dis. 11, 954–963. doi: 10.1093/clinids/11.6.954, PMID: 2690289

[B31] Sun T. Wu X. Cai Y. Zhai T. Huang L. Zhang Y. . (2021). Metagenomic next-generation sequencing for pathogenic diagnosis and antibiotic management of severe community-acquired pneumonia in immunocompromised adults. Front. Cell Infect. Microbiol. 11, 661589. doi: 10.3389/fcimb.2021.661589, PMID: 34141628 PMC8204719

[B32] Tang W. Zhang Y. Luo C. Zhou L. Zhang Z. Tang X. . (2021). Clinical application of metagenomic next-generation sequencing for suspected infections in patients with primary immunodeficiency disease. Front. Immunol. 12, 696403. doi: 10.3389/fimmu.2021.696403, PMID: 34484193 PMC8414648

[B33] Wang N. Liu J. Yang P. Fraidenburg D. Tang H. (2023). Editorial: Perspectives in pharmacological therapy targeting cellular metabolic pathways in respiratory diseases. Front. Pharmacol. 14, 1324586. doi: 10.3389/fphar.2023.1324586, PMID: 38089050 PMC10711599

[B34] Wang Y. Fan G. Horby P. Hayden F. Li Q. Wu Q. . (2019). Comparative outcomes of adults hospitalized with seasonal influenza A or B virus infection: application of the 7-category ordinal scale. Open Forum Infect. Dis. 6, ofz053. doi: 10.1093/ofid/ofz053, PMID: 30895200 PMC6419989

[B35] Winthrop K. L. Mariette X. Silva J. T. Benamu E. Calabrese L. H. Dumusc A. . (2018). ESCMID Study Group for Infections in Compromised Hosts (ESGICH) Consensus Document on the safety of targeted and biological therapies: an infectious diseases perspective (Soluble immune effector molecules [II]: agents targeting interleukins, immunoglobulins and complement factors). Clin. Microbiol. Infect. 24 Suppl 2, S21–S40. doi: 10.1016/j.cmi.2018.02.002, PMID: 29447987

[B36] Wolfe F. Caplan L. Michaud K. (2006). Treatment for rheumatoid arthritis and the risk of hospitalization for pneumonia: associations with prednisone, disease-modifying antirheumatic drugs, and anti-tumor necrosis factor therapy. Arthritis Rheum 54, 628–634. doi: 10.1002/art.21568, PMID: 16447241

[B37] Wu X. Sun T. He H. Xing L. Cheng Z. Geng S. . (2025). Effect of metagenomic next-generation sequencing on clinical outcomes of patients with severe community-acquired pneumonia in the ICU: A multicenter, randomized controlled trial. Chest 167, 362–373. doi: 10.1016/j.chest.2024.07.144, PMID: 39067508

[B38] Zhang P. Chen Y. Li S. Li C. Zhang S. Zheng W. . (2020). Metagenomic next-generation sequencing for the clinical diagnosis and prognosis of acute respiratory distress syndrome caused by severe pneumonia: a retrospective study. PeerJ 8, e9623. doi: 10.7717/peerj.9623, PMID: 32821543 PMC7395598

[B39] Zhang D. Chen S. Wang Y. Hou D. Chen C. Wang L. . (2022). The evaluation of next-generation sequencing assisted pathogenic detection in immunocompromised hosts with pulmonary infection: A retrospective study. Clin. Respir. J. 16, 793–801. doi: 10.1111/crj.13542, PMID: 36257289 PMC9716706

[B40] Zhou H. Larkin P. M. K. Zhao D. Ma Q. Yao Y. Wu X. . (2021). Clinical impact of metagenomic next-generation sequencing of bronchoalveolar lavage in the diagnosis and management of pneumonia: A multicenter prospective observational study. J. Mol. Diagn. 23, 1259–1268. doi: 10.1016/j.jmoldx.2021.06.007, PMID: 34197923

